# Heterologous Vector—mRNA Based SARS-CoV-2 Vaccination Strategy Appears Superior to a Homologous Vector—Based Vaccination Scheme in German Healthcare Workers Regarding Humoral SARS-CoV-2 Response Indicating a High Boosting Effect by mRNA Vaccines

**DOI:** 10.3390/vaccines11030701

**Published:** 2023-03-19

**Authors:** Catharina Gerhards, Margot Thiaucourt, Michael Hetjens, Verena Haselmann, Michael Neumaier, Maximilian Kittel

**Affiliations:** 1 Institute for Clinical Chemistry, Medical Faculty Mannheim, University of Heidelberg, Theodor Kutzer Ufer 1-3, 68167 Mannheim, Germany; 2 Department of Biomedical Informatics, Center for Preventive Medicine and Digital Health Baden-Württemberg, Medical Faculty Mannheim, Heidelberg University, 68167 Mannheim, Germany

**Keywords:** antibody dynamics, antibody kinetics, anti-SARS-CoV-2 antibodies, longitudinal assessment, serological immune response, humoral response post-vaccination, vaccination breakthrough, side effects, vaccination strategy

## Abstract

Background: Longitudinal humoral SARS-CoV-2 (severe acute respiratory syndrome coronavirus type 2) immunity for up to 15 months due to vaccination, the efficacy of vaccination strategies (homologous, vector–vector versus heterologous, vector–mRNA), the influence of vaccination side effects, and the infection rate in German healthcare workers need to be investigated. Methods: In this study, 103 individuals vaccinated against SARS-CoV-2 were enrolled to examine their anti-SARS-CoV-2 anti-N- and anti-RBD/S1-Ig levels. A total of 415 blood samples in lithium heparin tubes were prospectively obtained, and a structured survey regarding medical history, type of vaccine, and vaccination reactions was conducted. Results: All participants demonstrated a humoral immune response, among whom no values decreased below the positivity cutoff. Five to six months after the third vaccination, three participants showed anti-RBD/S1 antibodies of less than 1000 U/mL. We observed higher levels for heterologous mRNA-/vector-based combinations compared to pure vector-based vaccination after the second vaccination, which is harmonized after a third vaccination with the mRNA-vaccine only in both cohorts. The incidence of vaccine breakthrough in a highly exposed cohort was 60.3%. Conclusion: Sustained long-term humoral immunity was observed, indicating the superiority of a heterologous mRNA-/vector-based combination compared to pure vector-based vaccination. There was longevity of anti-RBD/S1 antibodies of at least 4 and up to 7 months without external stimulus. Regarding vaccination reactogenity, the occurrence of local symptoms as pain at the injection site was increased after the first mRNA application compared to the vector–vector cohort with a general decrease in adverse events at later vaccination time points. Overall, a correlation between the humoral vaccination response and vaccination side effects was not observed. Despite the high prevalence of vaccine breakthroughs, these only occurred in the later course of the study when more infectious variants, which are, however, associated with milder courses, were present. These results provide insights into vaccine-related serologic responses, and the study should be expanded using additional vaccine doses and novel variants in the future.

## 1. Introduction

The key strategy over the past year to overcome the global pandemic caused by severe acute respiratory syndrome coronavirus 2 (SARS-CoV-2) has been to provide vaccinations to stimulate the immune system prior to possible exposure to infection, thereby minimizing the extent of clinical manifestations [1]. Vaccination agents available in Germany are messenger RNA (mRNA)-based products such as BNT162b2 (Comirnaty, Pfizer-BioNTech, Mainz, Germany) and mRNA-1273 (Spikevax, Moderna) and vector-based products such as ChAdOx1 nCoV-19 (AstraZeneca, Cambridge, UK) and Ad16.COV.2.S (Janssen Pharmaceutical K.K.) [1,2,3]. Different vaccines are recommended depending on the medical history and age of the patient. In the course of the first immunization phase with the adeno-vector-based vaccine ChAdOx1 nCoV-19 (AstraZeneca), venous sinus thrombosis occurred, particularly in younger patients. Thus, vaccination guidelines were adjusted for patients younger than 30 years. The adaptations included a second vaccination with an mRNA-based vaccine, resulting in a heterologous vaccine combination [4]. Consequently, the population was divided into two cohorts: those who had received either homologous or heterologous vaccines for the first two vaccinations. Regarding the mentioned vaccination strategies, a homologous vaccination means that the same vaccine is administered twice (vector-based only), and a heterologous vaccination combines two different types of vaccines (mRNA- and vector-based). Due to the described adaption of vaccination guidelines, a combination of miscellaneous vaccines could have been used for the second and third vaccination in Germany. Therefore, different immune responses were reported in the described cohorts and should be investigated on the basis of humoral methods over a long time period. In general, the immune response can be assessed via both humoral and cellular systems. Thus, when antibody titers decrease, immunity can still be maintained at the cellular level. Regarding humoral SARS-CoV-2 immunity, the serological response can be assessed via antibodies directed against the nucleocapsid (N) protein and the spike (S) subunit 1 receptor-binding domain (RBD) of SARS-CoV-2 (RBD/S1). A disease-specific formation of antibodies can be detected by anti-N-abs. In contrast, both vaccination and COVID-19 can stimulate the production of anti-SARS-CoV-2-RBD/S1 abs. There are various epitopes against which antibodies can be formed, such as the receptor-binding domain (RBD) or the S1/S2 domain of the spike glycoprotein [5]. As described in previous studies, protective immunity mediated by antibodies with virus-neutralizing capacity correlates with anti-RBD/S1 antibodies [6,7,8]. Thus, anti-RBD/S1-pan-Ig serves as a surrogate parameter to monitor humoral SARS-CoV-2 immunity. For this reason, we focused only on antibody detection as an inexpensive and easy-to-implement assay in routine care [1,2,3].

A previous study including health workers who had been vaccinated with BNT162b2 (Comirnaty, Pfizer-BioNTech) revealed an initial increase in anti-RBD/S1 antibodies 2 weeks after the first dose. The maximum observed period was 5 weeks after study inclusion, leading to investigation via longitudinal follow-up. A further study examined a slightly longer period, up to 13 weeks after the first vaccination, and showed an increased anti-RBD/S1 antibody titer starting 3 weeks after the initial dose, which was exceeded after the second vaccination. Moreover, the researchers described a half-time decline in the anti-RBD/S1 antibody titer in a homologous vaccinated cohort 13 weeks after the initial dose, but did not assess the time for twofold decline using the heterologous strategy [9]. The dynamics and robustness of anti-RBD/S1 antibodies need to be investigated for homologous and heterologous vaccination strategies over a longer period, including the effects of the third vaccination. 

Evaluating the influence of various vaccination combinations, some studies have reported differences in the response of the humoral immune system [10,11]. Results have shown higher humoral reactogenicity from heterologous vaccination with the inactivated CoronaVac vaccine (Sinovac Biotech, Beijing, China) and the vector-based ChAdOx1 nCoV-19 vaccine (AstraZeneca), compared to the homogeneous CoronaVac strategy. These results should be extended to include combinations of mRNA- and vector-based vaccines. Additionally, by including a third vaccination in the analysis, more possible combinations could be investigated. In this regard, a previous study examined the efficacy against pathogens of booster strategies with the CoronaVac, ChAdOx1 nCoV-19, and NT162b2 vaccines, suggesting the superiority of heterologous combinations [9,10]. 

Therefore, long-term observations of more than 1 year, including a period without stimulation of up to 5.7 months after the third vaccination, need to be examined in order to investigate the robustness of antibody titers. A comparison of heterologous mRNA-/vector-based and homologous vector-based vaccination regimes is a central component of our study, as they affect a large number of highly exposed health care workers and the general public and, therefore, urgently need to be addressed in the long term. This refers to differences in antibody response, vaccination side effects, and the prevalence or timing of vaccination breakthroughs. Thus, in this study, we present the results of a longitudinal evaluation of antibody dynamics up to 15 months after the initial dose of the SARS-CoV-2 vaccine in healthy German workers. The aims of this study were as follows: (i) to examine anti-SARS-CoV-2 antibody titers, at six appointments to reveal vaccination-related humoral immune response in dependence of the vaccination strategy andantibody robustness 5 months after the third vaccination without external stimulation; (ii) to investigate the correlation between antibody titers and clinical vaccination reactions; and (iii) to illuminate the infection rates after vaccination, in the context of circulating variants.

## 2. Materials and Methods

### 2.1. Participant Recruitment and Sample Collection

Employees of the University Medical Center Mannheim, Germany, were invited to participate in the Immunitor-III study during the SARS-CoV-2 vaccine campaign. From February 2021 to June 2022, SARS-CoV-2 vaccinated individuals aged 18 years or older were enrolled in the study and underwent longitudinal blood sampling at six appointments. A secure web platform, Research Electronic Data Capture (REDCap), was utilized for the acquisition of standardized surveys addressing medical and personal data of participants at study entrance, and clinical data related to vaccination and SARS-CoV-2 status in the course of the study. Data collection was performed in pseudonymized form. The study was approved by the Institutional Review Board (2020-556N), informed consent was obtained from all participants, and the study was conducted in accordance with the Declaration of Helsinki.

**Figure 1:**
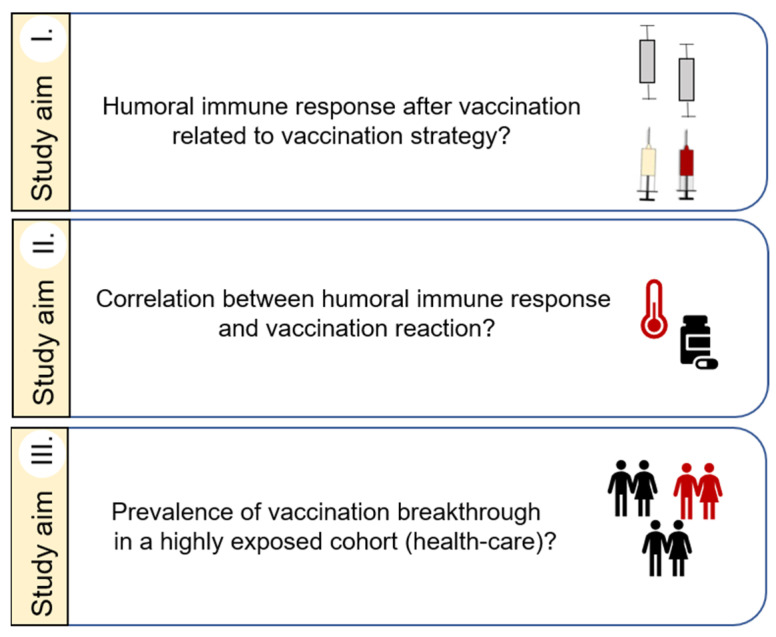
Study aims.

**Figure 2:**
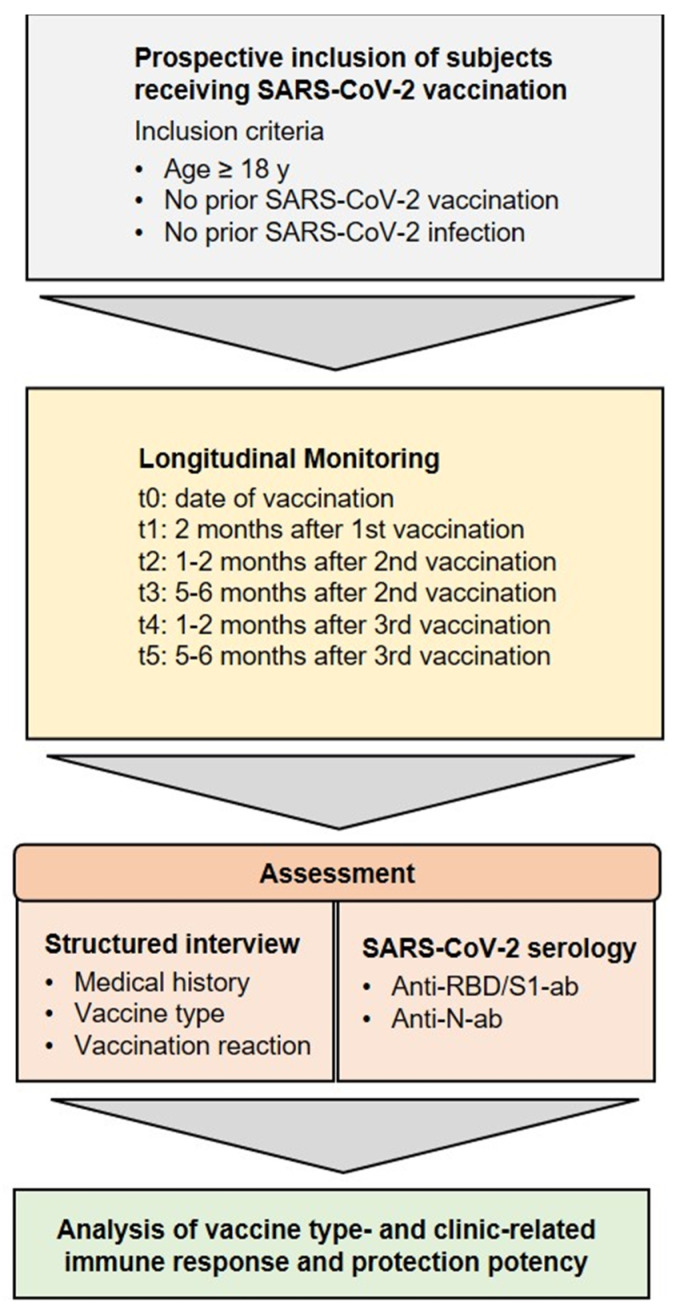
Inclusion criteria and analysis. Study concept and research objectives. Adults without a previous SARS-CoV-2 infection or vaccination were enrolled between February 2021 and June 2022 and were longitudinally assessed for clinical parameters and humoral immune responses.

For long-term surveillance of the humoral SARS-CoV-2 response and to carry out further analysis, blood samples were collected in 7.5 mL lithium heparin tubes (S-Monovette, Sarstedt AG & Co., Nümbrecht, Germany) at 6 time intervals: time of study enrollment (before or on the date of the first vaccination), 2 months after the first vaccination and before the second vaccination, 1.5 months after the second vaccination (6 weeks), up to 6 months after the second vaccination (24 weeks), 1.5 months after the third vaccination (7 weeks), and 6 months after the third vaccination (19 weeks) (for study protocol, see Figure 1 and Figure 2). Samples were centrifuged at 2000× *g* for 10 min at 18 °C, and plasma was aliquoted and stored at −80 °C until analysis. 

### 2.2. Anti-SARS-CoV-2 Antibody Detection 

The detection of SARS-CoV-2 anti-N and anti-RBD/S1 antibodies was performed using the same platform as that described in previous work [12]. For the detection of anti-SARS-CoV-2 anti-N Pan-Ig, a qualitative CE- and FDA-approved electrochemiluminescence immunoassay (ECLIA) with a positivity cut-off index (COI) ≥ 1.0 was used. The quantitative Elecsys^®^ Anti-SARS-CoV-2 S assay (Roche, Mannheim, Germany), similarly CE- and FDA-approved, was utilized to assess total anti-SARS-CoV-2 anti-RBD/S1 Ig, evaluated as reactive from a titer of 0.8 U/mL. After internal verification in line with DIN EN ISO 15189, the analyses were performed according to the manufacturer’s instructions at an accredited laboratory. 

### 2.3. General Data Analysis

All clinical data were systematically recorded by the REDCap platform (Vanderbilt University, Nashville, TN, USA). Statistical analyses were performed in Microsoft Excel 2019 (Microsoft, Redmond, WA, USA) and RStudio (version 4.1.2; RStudio, Boston, MA, USA) [9]. For all statistical analyses, a *p*-value < 0.05 was considered statistically significant and was indicated by an asterisk (*), and *p* < 0.01 was indicated by double asterisks (**). Non-normally distributed continuous variables were compared by the Kruskal–Wallis rank sum test, and for categorical variables, Fisher’s exact test was performed. Graphs were plotted using GraphPad Prism 7.05 (Dotmatics, Boston, MA, USA), RStudio (version 4.1.2; RStudio, Boston, MA, USA) [13], and Microsoft PowerPoint 2019 (Microsoft, Redmond, WA, USA).

## 3. Results

### 3.1. Demographics and Vaccination Strategies

A total of 103 participants were enrolled in the study, among whom 51 were included in longitudinal follow-up up to 13.5 ± 0.6 months after recruitment. During the 462 study days, 415 blood samples were collected in lithium heparin tubes and analyzed. Demographic data of the study cohort, including medical history and pre-existing medical conditions, are given in Table 1. Among the 103 participants, some individuals did not complete the structured questionnaire online or omitted particular questions, resulting in potential variation of the maximum participant size in the anamnestic data. Among the participants, 75.7% were female (f) (f = 78/103), the mean age was 39.64 ± 14.81 years, and the mean body mass index (BMI) was 25.32 (BMI range 15–50). At the initial interview, 24.7% were smokers, the majority of whom (7/20) smoked 11 to 20 cigarettes per day. At t_0_, one participant reported SARS-CoV-2 positivity in the past (1.2%). A total of 97% were health care workers, among whom 67 out of 82 reported being a medical professional. In terms of pre-existing diseases, 12.3% suffered from pulmonary disease, 7.4% from vascular disease, 3.7% from autoimmune disease, 2.5% from cancer in remission, and 1.2% from diabetes. Other diseases were also reported, the most frequent of which was hypothyroidism (6/17). A total of 40.7% took daily medication, and none were on immunosuppressive therapy. In this context, 9 out of 37 participants who presented allergies took antiallergic medication, and none of them were currently undergoing hyposensitization. Among the 50 participants who knew their blood type and rhesus factor, the majority indicated that it was 0+ (21%), followed by A+ (18.5%).

For the initial vaccination, 94.4% received the vector-based ChAdOx1 nCoV-19 (AstraZeneca), 4.4% received the mRNA-based NT162b2 (Comirnaty, Pfizer-BioNTech), and 1.1% received the mRNA-1273 (Spikevax, Moderna). For the second vaccination, the majority received the NT162b2 vaccine (65.5%), followed by ChA-dOx1 (32.1%) and mRNA-1273 (2.4%). Booster vaccination was performed exclusively with mRNA vaccines, predominantly NT162b2 (84.5%) (15.5% mRNA-1273). 

From these data, different vaccination strategies could be deduced, i.e., homologous vector-based or heterologous vector- and mRNA-based for the first and second vaccinations. However, for the third vaccination, only mRNA vaccines were used, resulting in a complete absence of purely vector-vaccinated participants at the t_4_ appointment. Regarding vaccine breakthroughs, during the spread of the Delta variant (B.1.617.2), one participant had a positive SARS-CoV-2 qPCR result (1.2%, *n* = 83). In spring 2022, during the expansion of the Omicron variant (B1.1.529), 31 of 84 participants (36.9%) experienced a vaccine breakthrough. Following the last appointment, vaccination breakthroughs were followed up, and SARS-CoV-2 positivity was reported in 44 of 73 participants (60.3%). All characteristics are summarized in more detail in Table 1.

### 3.2. Overall Antibody Trends

**Figure 3:**
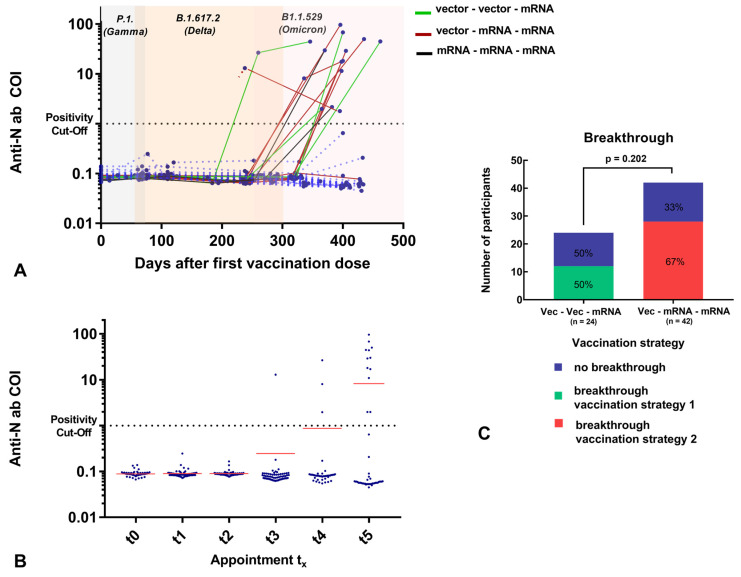
(**A**) Individual anti-N Ig titer (COI; *y*-axis) related to days after first vaccination (days; *x*-axis). Two values of one subject (titer of t_0_ = 0 days and t_3_ =238 days) were not connected due to missing data of t_1_ and t_2_, which could imply an earlier ab increase. The variants circulating in the respective period with a chronological overlap are highlighted in color (gray = P1. (Gamma), apricot = B.1.617.2 (Delta), light pink = B1.1.529 (Omicron)). Subjects with vaccination breakthrough before t_5_ are illustrated: Vector–vector–mRNA cohort: green, vector–mRNA–mRNA cohort: red, mRNA–mRNA –mRNA: black (individual cases). Most of the subjects experienced a breakthrough after the observation period (>t_5_). (**B**) Total anti-N Ig titer (COI; *y*-axis) at different time points (t_0_: date of vaccination; t_1_: 2 months after first vaccination; t_2_: 1–2 months after second vaccination; t_3_: 5–6 months after second vaccination; t_4_: 1–2 months after third vaccination; t_5_: 5–6 months after third vaccination (*x*-axis) for 101 participants (at time of study inclusion). Subjects with positive N-ab results at t_0_ were excluded from this analysis due to infection prior first vaccination. Mean anti-N-Ig titer is illustrated in red. (**C**): The percentage distribution of vaccination breakthroughs depending on the vaccination strategy is illustrated. Most of the participants were SARS-CoV-2-infected after t_5_ appoinment.

Initial blood sampling was performed before or on the same day as the first vaccination in order to establish a baseline value. Two participants were found to be seropositive for anti-SARS-CoV-2-anti-N and anti-SARS-CoV-2 anti-RBD/S1 antibodies; one had reported the COVID-19 disease, and one had a subclinical course. In addition, another individual who showed slightly positive anti-SARS-CoV-2 anti-RBD/S1 antibodies reported having a previous vaccination. From t_1_, an average of 2.3 months after the first vaccination but before the second vaccination, 98.8% of the participants (81/82) demonstrated anti-RBD/S1 positivity. In one seronegative case, however, there were only 5 days between vaccination and blood sampling. The median t_1_ titer was 71 U/mL (IQR 42, 112), a significant increase from t_0_ (*p* < 0.001). Furthermore, we observed an enormous increase at t_2_, 27 ± 9 days after the second vaccination, to a median of 7201 U/mL (IQR 1793, 10840) (*p* < 0.001). Antibodies significantly decreased to 784 U/mL (IQR 322, 1414) approximately 5.6 ± 0.7 months after the second vaccination (*p* < 0.001). Ten participants had already received their third vaccination prior to this appointment (4–38 days prior). This was the first appointment where anti-SARS-CoV-2-anti-N-ab positivity was observed that was not known at study inclusion, with a value of 12.9 COI. Three additional participants were tested for anti-SARS-CoV-2 anti-N-ab at t_4_ and 10 more at t_5_, 13.3 ± 0.5 months after study inclusion. The anti-SARS-CoV-2 anti-N-ab of the participant who was initially positive decreased from 101.8 to 51.2 COI, during the course of the study. Regarding all N-ab positive subjects a medium N-ab titer of 29.5 ± 27.5 COI was observed at t_5_ (*n* = 14). On average, there were 49.4 ± 33.1 days between the positive qPCR result and the sample collection. A total of 3 out of 15 participants did not have a positive qPCR or antigen test result, of whom one reported a high-risk contact, resulting in cold symptoms, and two had an inapparent course. Since most participants (*n* = 12/15) had a positive qPCR result between t_4_ and t_5_, no longitudinal assessment of N-ab is available. For two participants, a percentage increase to 195.2 ± 29% was observed between t_4_ and t_5_. One SARS-CoV-2 qPCR-positive participant did not attend the t_5_ appointment. The overall and individual development of N-ab is shown in Figure 3 and Supplemental Table S1. 

**Figure 4:**
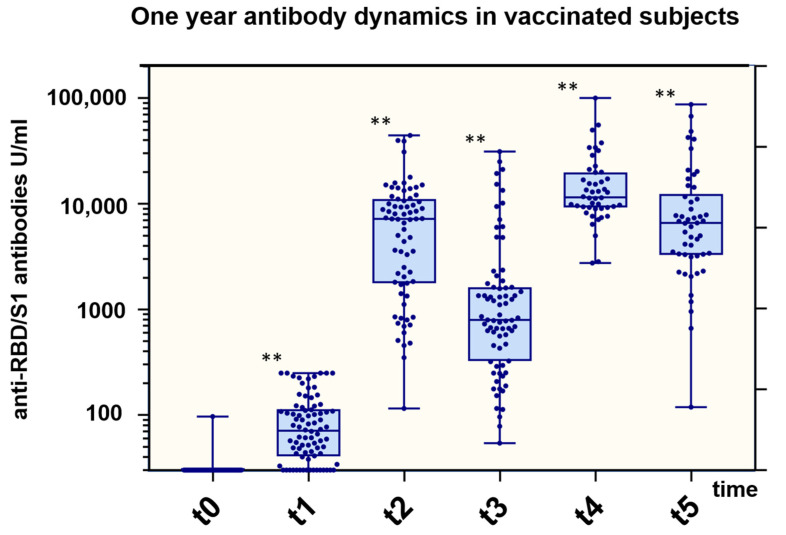
Total anti-RBD/S1 Ig titer related to vaccination dose. Boxplots for anti-RBD/S1 (U/mL; *y*-axis) at different time points (t_0_: date of vaccination; t_1_: 2 months after first vaccination; t_2_: 1–2 months after second vaccination; t_3_: 5–6 months after second vaccination; t_4_: 1–2 months after third vaccination; t_5_: 5–6 months after third vaccination (*x*-axis) for 100 participants (at time of study inclusion). In plots, interquartile ranges are shown in boxes with a line representing the median and whiskers representing the minimum and maximum data values. Dots indicate individual values. Antibody titers were compared to previous time points by the Kruskal–Wallis rank sum test. *p*-values < 0.05 were considered to be significant; ** *p*-values < 0.001.

The third vaccination resulted in a further significant increase in anti-SARS-CoV-2 anti-RBD/S1-ab to 11,490 U/mL (IQR 9239, 19,080) after 1.6 months. Finally, 4.6 months after the third vaccination, the titer decreased to a median of 6557 U/mL (IQR 3304, 11,506). During an interval without artificial stimulus, anti-SARS-CoV-2 anti-RBD/S1-ab decreased to 20 ± 17% between t_2_ and t_3_ (*n* = 58, 5.4 months after the second dose) and to 39 ± 15% between t_4_ and t_5_ (*n* = 28, 4.4 months after the third dose). Overall antibody dynamics are presented in Figure 4 and Table 2.

### 3.3. Anti-RBD/S1 Antibody Dynamics Correlated with Vaccination Strategy

**Figure 5:**
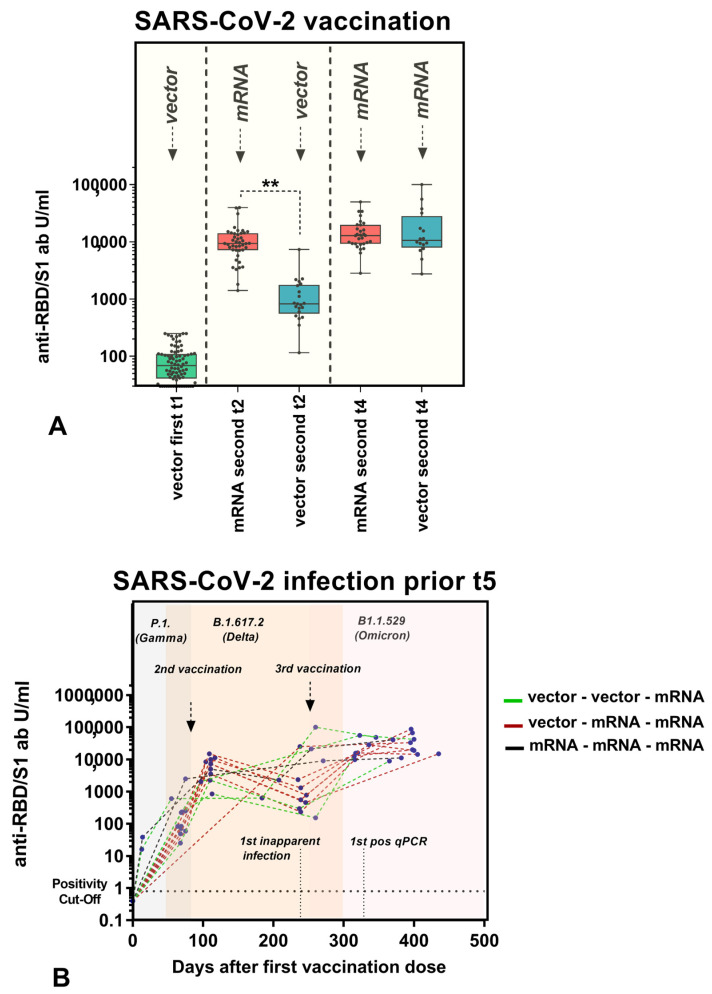
(**A**) Influence of vaccination strategy on antibody production. Dot plots illustrating anti-RBD/S1 antibodies (U/mL; *x*-axis) related to the vaccination schedule. All participants received ChAdOx1 nCoV-19 as the first vaccination. The homologous cohort received vector-based ChAdOx1 nCoV-19, and the heterologous cohort received mRNA-based NT162b2 as the second dose. This was reversed with the third vaccination, as all participants were vaccinated with NT162b2 or mRNA-1273. Antibody titers were compared between cohorts after the second and third vaccinations by the Kruskal–Wallis rank sum test. *p*-values 0.05 were considered to be significant; ** *p*-values < 0.001. (**B**) Individual anti-RBD/S1 Ig titer (U/mL; *y*-axis) related to days after the first vaccination (days; *x*-axis). The variants circulating in the respective period with a chronological overlap are highlighted in color (gray = P1. (Gamma), apricot = B.1.617.2 (Delta), light pink = B1.1.529 (Omicron)). Approximation of second/third vaccination, first inapparent infection and first positive qPCR result is illustrated. Vector–vector–mRNA cohort: green, vector–mRNA–mRNA cohort: red, mRNA –mRNA–mRNA: black (individual cases). Most of the subjects experienced a breakthrough after the observation period (t_5_).

As described, diverse subcohorts with different vaccine combinations were considered. In terms of follow-ups after the second vaccination, we compared two cohorts, hom1 and het1. Participants who received only mRNA-1273 (Spikevax, Moderna) or mRNA-based NT162b2 (Comirnaty, Pfizer-BioNTech) were excluded from the comparison due to the small number of cases in the study population. Thus, since all participants in this comparison received ChAdOx1 (AstraZeneca) as their first vaccine, those who received mRNA-based NT162b2 (Comirnaty, Pfizer-BioNTech) for the second vaccination were assigned to het1, and those who received ChAdOx1 (AstraZeneca) were assigned to hom1. The baseline values of both cohorts were comparable due to the use of identical vaccines (*p* = 0.163). In contrast, significantly higher titers were detected in participants who received heterologous vaccinations, with a median of 9378.50 U/mL (7180.00, 14,103.00), compared to 826.30 U/mL (600.90, 1711.00) (*p* < 0.001) at t_2_. Up to 6.9 months after the second vaccination, the heterologous cohort still showed a tendency towards higher anti-RBD/S1-abs compared to the homologous vector-vaccinated group (1999 ± 3827 U/mL versus 495 ± 1034 U/mL, *p* = 0.102). After the third vaccination, the participants who previously received heterologous ChAdOx1/mRNA-based NT162b2 were categorized as hom2 and those who received homologous ChAdOx1 as het2, as the booster vaccination was administered with mRNA-based vaccines only. Convergence of the median titers of both cohorts was observed, with a median value of 12,852 (9,308, 17,567.25) in hom2 and 10,582 (8,592.25, 20,863) in het2 (*p* = 0.714). All cohort-related titers are illustrated in Figure 5A. Moreover, Figure 5B shows the individual anti-RBD/S1 abs of subjects infected during the observation phase (before t_5_) related to the vaccination strategy. A reference to the second and third vaccination (the first vaccination was day 0) and the circulating SARS-CoV-2 variants are presented. Moreover, we have indicated the approximate time of the first inapparent course (no positive test) and the first positive qPCR result, in order to allow an estimation of when the vaccination effect is supplemented by an infection stimulus.

### 3.4. Anti-RBD/S1 Antibody Dynamics Correlated with Vaccination Reaction

**Figure 6:**
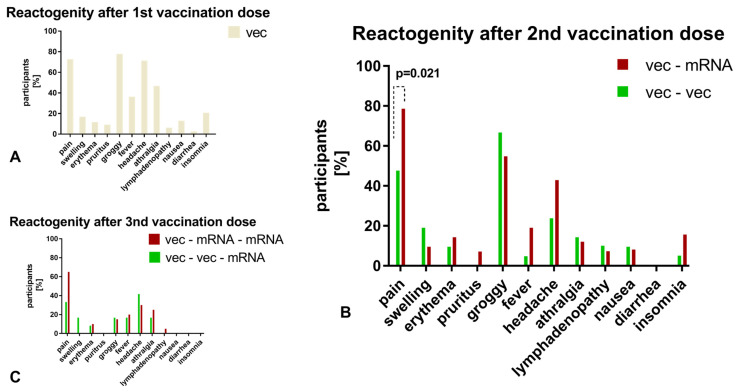
(**A**): For the first vaccination, only symptoms after the vector-based immunization are illustrated due to a small number of mRNA-vaccinated participants. (**B**,**C**): Depending on the vaccination scheme (homologous versus heterologous), the vaccination reactions are compared after the second vaccination. Only significant differences are indicated by a *p*-value. Vector–vector/+mRNA cohort: green, vector–mRNA/+mRNA cohort: red.

We considered the humoral response to vaccination separately for participants with and without early vaccination reactions. With regard to the first vaccination, only 9% of participants (*n* = 7) showed no reaction, and among those who were symptomatic, 85% felt groggy (*n* = 63), 80% reported localized pain (*n* = 59), and 77% experienced a headache (*n* = 57). Anti-SARS-CoV-2 anti-RBD-ab did not differ in the cohorts, with values of 39.70 U/mL (21.84, 89.92) for the asymptomatic cohort and 72.60 U/mL (43.52, 119.35) for the symptomatic cohort (*p* = 0.160). Only 5 subjects presented with only local symptoms, while 69 presented with systemic symptoms, and no difference was observed in anti-SARS-CoV-2 anti-RBD-ab titers (systemic: 72.51 U/mL (43.12, 115.65); local: 99.45 U/mL (70.43, 121.80); *p* = 0.575). Regarding the second vaccination, 7 participants reported no symptoms and 57 described symptoms, among which itching was the most common (95%), followed by swelling and erythema (86%) and pain at the site of injection (77%). The anti-SARS-CoV-2 anti-RBD-ab titer, 1.4 months after the second vaccination, was not affected by the appearance of symptoms (6175 U/mL (1889.75, 8967) versus 7158 U/mL (1710, 10,790.75); *p* = 0.878). In addition, among symptomatic participants, those with systemic symptoms (*n* = 45) did not have higher titers than those with local symptoms (*n* = 12) (*p* = 0.422). After the third vaccination, 34/51 participants reported experiencing a reaction. However, we did not observe any significant effect on the antibody level (9880 U/mL (8828.50, 13,054.25) versus 12,780 U/mL (9001, 19,885); *p* = 0.183). With regard to the reactogenicity depending on the vaccination strategy, a higher incidence of local pain was found in the heterologous cohort after the second vaccination (*p* = 0.021). The vaccination reactions after the first, second, and third vaccination are illustrated in Figure 6. A comparison of homologous vector–vector and heterologous vector–mRNA vaccination strategy was compared after the second vaccination.

## 4. Discussion

COVID-19 vaccines play a crucial role in the fight against new variants of concern. They provide protection against severe illness and hospitalization, reducing the burden on the healthcare system [14]. By reducing the number of people who become infected with COVID-19, vaccines help to slow the spread of the virus, including new variants. It is possible that new variants could reduce the efficacy of existing vaccines [15]. In particular, the occurrence of unpredictable, potentially life-threatening adverse events has led to the unanticipated use of heterologous vaccination regimens [16].

In this study, we investigated the long-term humoral immune response to SARS-CoV-2 vaccination as a function of different vaccine combinations and individual clinical contexts. This is of current interest, as other studies have already shown that a combination can ensure unusually strong stimulation of the immune system [1,2,3,17].

Numerous publications have been published on heterologous vaccine regimens and their immunogenic activity. A strong humoral and cellular immunological response has been proven in animal experiments, indicating a high protective effect [18]. Some studies have described the humoral response to heterologous vaccination in the form of a prospective longitudinal study. Wanlapakorn et al. were able to demonstrate the superiority of a heterologous vaccination approach based on a vector-based vaccine combined with attenuated viral proteins [11,19]. Likewise, it was proven that heterologous vaccination regimens are superior in terms of the antibody titer level that develops in response to so-called booster vaccinations [20,21]. Interestingly, a comparison of our data with other publications indicates that the sequence of administration of the mRNA vaccine and the vaccine manufacturer are of secondary importance. However, this superiority of a heterologous combination refers only to subjects with first vector-based vaccination compared with double-vector vaccination and should also be compared with pure mRNA vaccinated subjects in follow-up studies. Thus, in contrast to the studies reported above, we distinguished between homologous and heterologous vaccines in terms of mRNA- and vector-based agents in the second vaccination. However, anti-SARS-CoV-2 anti-RBD-ab levels were significantly higher in our heterologous cohort, administered BNT162b2 as the second dose, than in the cohort administered only ChAdOx1nCov-19 (*p* < 0.001). Moreover, considering the anti-RBD/S-antibody levels up to 6.9 months without stimulus after the second vaccination, tendencies for a higher titer in the heterogeneous vaccine combination was observed (*p* = 0.102). This might indicate prolonged longevity of the antibodies, presumably due to a higher initial titer level. In the longitudinal assessment, anti-SARS-CoV-2 anti-RBD-ab titers were equalized after the booster vaccination in both cohorts. Since the titer converged after the third vaccination, when everyone had received at least one mRNA vaccination, there was some indication that this significant booster effect by an mRNA vaccine is possible after both the second and third vaccination. It should be evaluated whether, in resource-limited settings, a heterologous vaccine regimen with two different vaccines and two administration points would be preferable to a regimen with two homologous vaccines and one booster. This would not only alleviate the financial and workforce burden but also allow vaccination campaigns to be accelerated [22,23].

Supplementing the effect of greater antibody titers achieved by the heterologous vaccination regimen, respectively, a high boosting effect of vector-vaccinated subjects via the application of an mRNA vaccine, as described in this article, it is important to emphasize that other studies have already demonstrated that adenovirus vector-based SARS-CoV-2 vaccines lead to a more modest titer development compared to mRNA-based vaccines. For instance, in their comparison of different booster vaccination regimens, Atmar et al. were able to demonstrate a higher degree of immunogenicity in mRNA vaccines with respect to their neutralization capacity [24]. Differentiating from our study, it has to be stated that, in contrast to our approach, homologous basic immunization with different booster vaccines was compared. The comparison of the antibody formation effect in heterologous basic vaccination compared to other published studies indicates that this effect is significantly more prominent [25,26].

Due to public concerns that vaccination reactions are only associated with the development of protective antibodies, those were evaluated in the clinical context [27]. Evaluation [25,26] of this hypothesis indicated no significant correlation between the occurrence of a vaccination reaction and antibody development. Nevertheless, it should be pointed out that a high proportion of study participants (91%) reported a reaction after the first vaccination, but they had no history of reactions to other vaccinations. The most common symptom was a feeling of light headedness, followed by local pain at the injection site, and headache at administration. Notably, systemic reactions have been reported for vector-based vaccines and were confirmed in our study [28]. Regarding the second vaccination, the most predominant symptom was a local reaction with pain at the injection site, reported by 77% of participants.. Considering the reactogenicity in dependence of the vaccine administration, this might be explained due to a higher existence of local symptoms as pain at the injection site caused by mRNA vaccines. These data are in agreement with the results of other observational studies. A correlation between antibody formation and vaccination response was not demonstrated at any vaccination time point. Furthermore, in both our study and others, a decrease in adverse events at later vaccination time points was observed [29].

In addition to antibody development, within the cohort of middle-aged healthcare professionals, we further investigated the persistence of antibody titers in the absence of an external stimulus, such as vaccination or infection. Glöckner et al. described a significant decrease in anti-SARS-CoV-2 anti-RBD-ab at 13 weeks after the third vaccination [9]. In this study, for a mean observation period of 19 weeks after the second vaccination, a significant decrease in antibody titers with persistent seropositivity was observed in all 58 participants, excluding those with a confirmed breakthrough infection. Sugiyama et al. proposed an anti-SARS-CoV-2 anti-RBD-ab titer below 338 BAU/mL as a possible cutoff for protection against a severe variant infection [30]. In our long-term cohort, assessed up to 5.7 months after the third vaccination, 94% of participants showed titers higher than 1000 U/mL, and only one participant had a value less than 338 U/mL [31]. This indicates a long-lasting immunological response due to the heterologous vaccination strategy. The decline in antibody titers as well as neutralizing activity in plasma is a physiological phenomenon. Nevertheless, this is not immediately associated with higher susceptibility to severe infection since RBD-specific memory B cells are persistent [32].

According to previous studies [33,34], it is important to clarify that the analytical assays used in this study to assess humoral immunity are considered to be surrogate markers. Although several studies have addressed the correlation between virus neutralizing assays as the gold standard and anti-SARS-CoV-2 anti-RBD-ab assays, the authors suggest a value higher than 2000 BAU/mL as a possible good surrogate parameter for the prediction of immunity [35]. This suggests a limitation of serological assays due to the limited comparability among manufacturers. Therefore, we recommend using the same methodology for longitudinal monitoring of patients. Besides the lack of harmonization, the recommended cutoff values might be problematic in the context of emerging novel virus variants of concern [36]. Only if these limitations are rigorously addressed can they be used as a cost-effective surrogate marker for virus neutralization capacity [7,37]. T cell-mediated immune responses provide the other essential branch of the immune defenses in targeting intracellular pathogens [33,34]. For example, T-cell responses have been demonstrated to play a critical role in viral elimination during infection with influenza viruses or SARS-CoV. This cellular immune response is both stimulated by infection and by the vaccines that were evaluated in this study. It could be shown that the cellular and humoral immune response correlate predominantly well [38]. However, it must be mentioned that the cellular immune response is significantly more robust against viral mutations and is almost not affected by the exchange of single amino acids [39]. A very special case, justifying the much more complex investigation of cellular immune responses, is the examination of vaccinated subjects without detectable antibodies [40]. Since these constellations did not occur in the study collective, no cellular immunity investigation was carried out.

The occurrence of breakthrough infections during the 15-month observation period in this high-risk group due to elevated exposure is another aspect of the research addressed in the study. Nearly all breakthrough infections observed were attributable to the first Omicron variant (B.1.1.529/BA.1). It is not possible to draw a conclusion regarding the protective efficacy of vaccine combinations due to the relatively heterogeneous study population and the limited number of cases. However, despite the high number of breakthrough infections (60.3%; 44/73), no severe disease courses were reported. We did not observe a significant difference in the breakthrough rate between the “vector–mRNA–mRNA” and the “vector–vector–mRNA cohort”. In contrast to only considering the vaccination breakthrough rates, it is common to evaluate the efficacy of vaccines based on hospitalization rates [41]. In this regard, further large-scale studies would be required to clarify whether the significantly higher humoral immune response to heterologous vaccine combinations could be associated with an increased efficacy of the virus neutralization capability. Furthermore, when considering the subcollective of breakthrough infections, it should be noted that the symptoms were very moderate, despite existing comorbidities as well as the absence of COVID-associated hospitalizations. These observations are in line with other published data, which state that a high antibody titer is not only associated with a probably higher protection against infection but also with a significantly reduced level of symptoms [42,43]. In addition, almost all of the SARS-CoV-2 infections occurred during the circulation of the Omicron variant. This may at least indicate that although variants with higher infectivity can lead to breakthroughs, infections with variants that cause more severe courses might be avoided in our highly exposed observation cohort.

However, several limitations of the data need to be critically considered. In order to make a stronger statement about the humoral immune response in relation to the vaccine combination, the inclusion of a control cohort with a homologous mRNA vaccine regimen would have been of great value. Nevertheless, the predominance of a heterologous mRNA/vector-based vaccine combination compared with vector-based vaccination alone can be proven in terms of antibody titers. Regrettably, individuals without an early vaccination response were significantly under-represented, limiting the power of the null hypothesis. One important aspect to emphasize is that the use of a combination of vaccines is an approach that has not been investigated by any manufacturer in a large-scale controlled registration program. The majority of the available scientific data is based on observational studies. Therefore, it cannot be ruled out that there may be adverse effects that have not yet been identified due to the lack of structured surveillance. In addition, a survey of individual COVID-19 symptoms of all vaccine breakthroughs could not be performed in comparable detail to the survey of vaccine reactions, because most experienced illnesses after the observation period. This resulted in more open responses indicating mild COVID-19 courses that could have been implemented more systematically if the study design had been longer. An additional limitation concerns the assays used to assess immunity. Even though the methods used in this study are well researched and have high congruence, virus neutralization assays are considered the gold standard for the assessment of the humoral immune capacity. Assays that assess cellular immunity would complement these methods and should be addressed in follow-up projects. Despite these limitations, the authors consider that important questions in the study were answered, and insights can be gained regarding the humoral antibody response after vaccinations, depending on the clinic and vaccine combination.

In conclusion, we were able to demonstrate persistent humoral antibody titers up to 15 months after the initial vaccination, suggesting that a heterologous mRNA/vector combination is preferable to a vector vaccination only. For this combination, the sequence of vaccines is not likely to be important, as we observed antibody titer similarity between the two vaccination regimens, indicating a high booster effect by mRNA vaccines as second or third dose. A correlation between the humoral vaccination response and early local or systemic reactions was not observed. Furthermore, the humoral response was long-lasting over an observation period of at least 4 months, but up to 7 months in the absence of an external stimulus. The data indicate evidence of protection against infection with a strict use of the assessed vaccination regimes. These results provide insight into vaccine-related serologic responses, and the study could be expanded in the future with respect to additional vaccine doses and new variants.

## Figures and Tables

**Table 1:** Patient demographics and clinical history.

Variable	Overall
	*n* = 103
**Demographics**	
Sex F/M (%)	78/25 (76.7/24.3)
Age (mean (SD))	39.64 (14.81)
BMI (mean (SD))	25.32 (5.10)
Smoking	20/81 (24.7%)
1–5 cigarettes/day	4 (4.9%)
6–10 cigarettes/day	6 (7.4%)
11–20 cigarettes/day	7 (8.6%)
31–40 cigarettes/day	3 (3.7%)
**Pre-existing disease**	
Pulmonary disease	10/81 (12.3%)
Vascular disease	6/81 (7.4%)
Autoimmune disease	3/81 (3.7%)
Cancer (in remission)	2/81 (2.5%)
Diabetes	1/81 (1.2%)
Other disease	17/81 (21.0%)
Hypothyroidism	6/81 (7.4%)
Allergy	37/81 (45.7%)
**Medication**	
Any medication	33/81 (40.7%)
Immunosuppression	0/81 (0%)
Antiallergic medication	9/81 (11.1%)
**Blood type**	
Unknown	31 (38.3%)
0+	17 (21.0%)
A+	15 (18.5%)
B+	3 (3.7%)
AB+	4 (4.9%)
0-	4 (4.9%)
A-	6 (7.4%)
AB-	1 (1.2%)
**SARS-CoV-2 anamnesis**	
Contact (1st vaccination)	13/82 (15.9%)
Previous infection	2/103 (1.9%)
**SARS-CoV-2 vaccination**	
**First dose**	
ChAdOx1 nCoV-19 (AstraZeneca)	85 (94.4%)
BNT162b2 (Pfizer-BioNTech)	4 (4.4%)
mRNA-1273 (Moderna)	1 (1.1%)
**Second dose**	
ChAdOx1 nCoV-19 (AstraZeneca)	27 (32.1%)
BNT162b2 (Pfizer-BioNTech)	55 (65.5%)
mRNA-1273 (Moderna)	2 (2.4%)
**Third dose**	
ChAdOx1 nCoV-19 (AstraZeneca)	0 (0%)
BNT162b2 (Pfizer-BioNTech)	59 (84.5%)
mRNA-1273 (Moderna)	11 (15.5%)
**Vaccination strategy**	
**First and second dose**	
Homologous (AstraZeneca)	26 (31.0%)
Heterologous (AstraZeneca/ BioNTech)	53 (63.1%)
Homologous (BioNTech)	4 (4.8%)
Homologous (Moderna)	1 (1.2%)
**Second and third dose**	
Homologous (mRNA vaccines)	47 (66.2%)
Heterologous (mRNA/vector)	24 (33.8%)

**Table 2:** SARS-CoV-2 antibodies.

Variable	Overall
t_0_ anti-N abs (median (IQR))	0.09 (0.09, 0.09)
t_1_ anti-N abs (mean (SD))	0.94 (7.70)
t_2_ anti-N abs (median (IQR))	0.09 (0.09, 0.09)
t_3_ anti-N abs (median (IQR))	0.07 (0.07, 0.09)
t_4_ anti-N abs (median (IQR))	0.08 (0.08, 0.09)
t_5_ anti-N abs (median (IQR))	0.06 (0.06, 1.50)
t_0_ anti-RBD/S1 (median (IQR))	0.40 (0.40, 0.40)
t_1_ anti-RBD/S1 (median (IQR))	71.43 (41.76, 111.68)
t_2_ anti-RBD/S1 (median (IQR))	7201.00 (1793.00, 10,839.50)
t_3_ anti-RBD/S1 (median (IQR))	791.70 (376.95, 1619.00)
t_4_ anti-RBD/S1 (median (IQR))	11,490.00 (9239.00, 19,079.50)
t_5_ anti-RBD/S1 (median (IQR))	6557.00 (3304.25, 11,506.25)

## Data Availability

The data presented in this study are available on request from the corresponding author.

## Supplementary Materials

The following supporting information can be downloaded at: https://www.mdpi.com/article/10.3390/vaccines11030701/s1, Table S1: Individual Breakthrough data.

## Abbreviations

Ab	Antibody
BMI	Body mass index
CE	Communauté Européenne
COI	Cut-off index
COVID-19	Coronavirus disease 2019
ECLIA	Electrochemiluminescence immunoassay
FDA	Food and Drug Administration
Ig	Immunoglobulin
ISO	International Organization for Standardization
qPCR	Quantitative polymerase chain reaction
RBD	Receptor-binding domain
REDCap	Research Electronic Data Capture
mRNA	Messenger ribonucleic acid
S	Spike protein
SARS-CoV-2	Severe acute respiratory syndrome coronavirus 2
