# Supplementary material for: Heterologous Vector—mRNA Based SARS-CoV-2 Vaccination Strategy Appears Superior to a Homologous Vector—Based Vaccination Scheme in German Healthcare Workers Regarding Humoral SARS-CoV-2 Response Indicating a High Boosting Effect by mRNA Vaccines
Source: Vaccines (Basel). 2023 Mar 19;11(3):701. doi: 10.3390/vaccines11030701 (PMC10054089; doi:10.3390/vaccines11030701)
Supplement: Supplementary file 1 [file vaccines-11-00701-s001.zip › vaccines-2268799-supplementary.pdf]

vaccines

Supplemental Table S1: Individual Breakthrough data.

No.	Break-through	Days [pos qPCR - sampling]	Anti-N-ab t4	Anti-N-ab t5	Anti-S/RBD-ab t4	Anti-S/RBD-ab t5	Circulating variant	Vaccination strategy
1	pos	t5=47	0.085	0.207	9548	7361	B1.1.529	vec - vec - mRNA
2	pos	t prior to qPCR	Na	0.045	Na	1182	B1.1.529	vec - mRNA -mRNA
3	pos	t prior to qPCR	0.081	0.057	16857	6853	B1.1.529	vec - mRNA -mRNA
4	pos	t prior to qPCR	Na	0.059	Na	3326	B1.1.529	vec - mRNA -mRNA
5	pos	t prior to qPCR	0.09	0.06	9242	3203	B1.1.529	vec - mRNA -mRNA
6	pos	t prior to qPCR	0.079	Na	10148	Na	B1.1.529	vec - mRNA -mRNA
7	neg	Na	0.07	Na	11355	Na	B1.1.529	vec - vec - mRNA
8	pos	t5=20	0.076	28.8	16091	14277	B1.1.529	vec - mRNA -mRNA
9	pos	t prior to qPCR	Na	Na	Na	Na	B1.1.529	vec - mRNA -mRNA
10	neg	Na	Na	Na	Na	Na	B1.1.529	vec - vec - mRNA
13	neg	Na	0.085	0.055	8197	3397	B1.1.529	vec - mRNA -mRNA
14	neg	Na	0.103	0.077	34049	7534	B1.1.529	vec - mRNA -mRNA
15	pos	t5=99	Na	49.74	Na	14881	B1.1.529	vec - mRNA -mRNA
17	pos	t prior to qPCR	Na	Na	Na	Na	B1.1.529	vec - mRNA -mRNA
20	neg	Na	0.063	0.059	31780	1355	B1.1.529	vec - vec - mRNA
22	pos	t prior to qPCR	Na	Na	Na	Na	B1.1.529	vec - vec - mRNA
23	neg	Na	0.082	0.06	2736	658,9	B1.1.529	vec - vec - mRNA
25	neg	Na	0.081	0.057	9695	3297	B1.1.529	vec - mRNA -mRNA
26	neg	Na	Na	0.057	Na	4627	B1.1.529	vec - mRNA -mRNA
27	pos	t prior to qPCR	Na	Na	Na	Na	B1.1.529	Na
28	pos	t prior to qPCR	Na	Na	Na	Na	B1.1.529	Na
30	pos	Inapparent	Na	1.79	Na	33184	B1.1.529	vec - mRNA -mRNA
31	neg	Na	Na	Na	Na	Na	B1.1.529	vec - mRNA -mRNA
32	neg	Na	0.084	0.057	11625	7698	B1.1.529	vec - mRNA -mRNA
34	pos	t prior to qPCR	Na	Na	Na	Na	B1.1.529	vec - vec - mRNA
36	pos	t5=78	0.082	11.42	9824	67025	B1.1.529	vec - mRNA -mRNA
37	neg	Na	Na	0.053	Na	4045	B1.1.529	vec - mRNA -mRNA
38	pos	t4= 18d; t5=82d	8.11	18.18	28632	18952	B.1.617.2/ B1.1.529	vec - mRNA -mRNA
39	pos	t5=105	0.084	44.72	15520	10384	B1.1.529	vec - vec - mRNA
40	neg	Na	Na	0.06	Na	119	B1.1.529	vec - vec - mRNA
42	neg	Na	0.083	0.056	9936	4812	B1.1.529	vec - vec - mRNA
43	neg	Na	Na	Na	Na	Na	B1.1.529	vec - mRNA -mRNA
44	neg	Na	0.081	0.061	9382	4962	B1.1.529	vec - vec - mRNA
48	neg	Na	0.058	Na	13670	Na	B1.1.529	vec - mRNA -mRNA
49	pos	t prior to qPCR	0.059	0.053	13142	6071	B1.1.529	vec - mRNA -mRNA
50	pos	t prior to qPCR	Na	Na	Na	Na	B1.1.529	Na
51	neg	Na	0.087	0.055	2835	956,1	B1.1.529	vec - mRNA -mRNA
52	pos	t prior to qPCR	Na	Na	Na	Na	B1.1.529	Na
54	pos	t prior to qPCR	0.06	0.057	4956	2247	B1.1.529	vec - vec - mRNA
55	pos	Inapparent	26.56	44.14	100000	48216	B1.1.529	vec - vec - mRNA
57	pos	t prior to qPCR	0.082	Na	33901	Na	B1.1.529	vec - mRNA -mRNA

58	pos	t prior to qPCR	Na	Na	Na	Na	B1.1.529	vec - vec - mRNA
59	neg	Na	Na	0.057	Na	3133	B1.1.529	vec - mRNA -mRNA
60	pos	t5=43	0.079	17.43	12780	20177	B1.1.529	vec - mRNA -mRNA
61	neg	Na	Na	51.0	Na	20828	B1.1.529	vec - mRNA -mRNA
62	pos	t prior to qPCR	Na	Na	Na	Na	B1.1.529	vec - mRNA -mRNA
63	neg	Na	0.066	0.066	11228	5380	B1.1.529	vec - vec - mRNA
66	neg	Na	0.081	0.054	19885	7528	B1.1.529	vec - mRNA -mRNA
67	pos	t prior to qPCR	0.09	0.07	11327	2187	B1.1.529	vec - mRNA -mRNA
69	neg	Na	0.08	0.053	37654	11652	B1.1.529	vec - vec - mRNA
71	pos	t prior to qPCR	Na	Na	Na	Na	B1.1.529	vec - vec - mRNA
72	neg	Na	0.085	0.06	9330	2295	B1.1.529	vec - mRNA -mRNA
73	pos	t5=54	Na	96.3	Na	87242	B1.1.529	vec - mRNA -mRNA
75	pos	t prior to qPCR	0.085	0.054	15283	6837	B1.1.529	vec - mRNA -mRNA
76	pos	t prior to qPCR	Na	Na	Na	Na	B1.1.529	vec - mRNA -mRNA
77	neg	Na	Na	0.09	Na	4687	B1.1.529	vec - vec - mRNA
78	pos	t prior to qPCR	Na	Na	Na	Na	B1.1.529	vec - mRNA -mRNA
79	pos	t prior to qPCR	0.055	0.054	19698	6537	B1.1.529	vec - mRNA -mRNA
81	pos	t4=4d, t5 Na	1.97	Na	8927	Na	B1.1.529	vec - vec - mRNA
83	pos	t5=9	0.079	0.645	7077	17170	B1.1.529	vec - vec - mRNA
84	neg	Na	Na	Na	Na	Na	B1.1.529	vec - mRNA -mRNA
86	pos	t prior to qPCR	Na	Na	Na	Na	B1.1.529	vec - mRNA -mRNA
88	neg	Na	0.08	0.05	7416	2046	B1.1.529	vec - mRNA -mRNA
89	pos	t prior to qPCR	Na	Na	Na	Na	B1.1.529	vec - vec - mRNA
90	neg	Na	0.081	Na	49880	Na	B1.1.529	vec - mRNA -mRNA
91	neg	Na	0.066	0.053	13445	6577	B1.1.529	vec - mRNA -mRNA
92	neg	Na	0.062	Na	7588	Na	B1.1.529	vec - vec - mRNA
93	pos	t5=43d	0.08	67.81	55724	42426	B1.1.529	vec - vec - mRNA
96	pos	t prior to qPCR	0.086	0.061	12924	3505	B1.1.529	vec - mRNA -mRNA
97	neg	Na	0.084	0.052	17224	8875	B1.1.529	vec - vec - mRNA
98	pos	t5=28	0.073	29.65	21093	40855	B1.1.529	mRNA - mRNA -mRNA
99	pos	t prior to qPCR	Na	Na	Na	Na	B1.1.529	mRNA - mRNA -mRNA
101	pos	t5=24	0.079	2.15	9001	11069	B1.1.529	mRNA - mRNA -mRNA
